# Evaluation of Tilt and Decentration of Intraocular Lens After Combined Femtosecond Laser‐Assisted Cataract Surgery and Vitrectomy

**DOI:** 10.1155/joph/6845362

**Published:** 2026-07-09

**Authors:** Tomoyuki Watanabe, Takeru Tuboi, Koki Honzawa, Hiroshi Horiguchi, Akira Watanabe, Tadashi Nakano

**Affiliations:** ^1^ Department of Ophthalmology, The Jikei University School of Medicine, 3-25-8 Nishi-shimbashi, Minato-ku, 105-8461, Tokyo, Japan, tokyo.ac.jp

**Keywords:** decentration, femtosecond laser-assisted cataract surgery, gas tamponade, intraocular lens, tilt, vitrectomy

## Abstract

**Purpose:**

To evaluate the changes in tilt and decentration prior to and following microincision vitrectomy and femtosecond laser‐assisted cataract surgery (FLACS) using a 7 mm intraocular lens (IOL).

**Methods:**

Patients were grouped by surgical method: conventional phacoemulsification surgery and vitrectomy without gas (Group A: 27 eyes), conventional phacoemulsification surgery and vitrectomy with gas (Group B: 72 eyes), FLACS and vitrectomy without gas (Group C: 29 eyes), and FLACS and vitrectomy with gas (Group D: 25 eyes). In the femtosecond‐laser group, a 5.6 mm capsulotomy was created. 7 mm IOL was inserted in all cases. IOL tilt and decentration were compared using swept‐source anterior segment optical coherence tomography for up to 12 months postoperatively.

**Results:**

On postoperative Day 1, the IOL tilt increased in all groups compared to the preoperative values, with a significantly greater increase observed in Groups B and D. Moreover, in Groups B and D, the tilt decreased substantially after Day 1 and gradually declined at 12 months. Similarly, Groups A and C also showed a decrease in tilt after Day 1, with a gradual decline at 12 months. Groups B and D showed greater initial decentration, which decreased over time. In contrast, Groups A and C exhibited more stable decentration values throughout the follow‐up period.

**Conclusion:**

In combined cataract surgery and vitrectomy, no difference was observed in the tilt and decentration of the 7 mm IOL between 5.6 mm anterior capsulotomy created by FLACS and continuous curvilinear capsulorhexis, regardless of gas tamponade. Combining FLACS with vitrectomy is an equivalent technique when combined with conventional phacoemulsification surgery and vitrectomy is performed.

## 1. Introduction

Since the initial report of combined cataract and vitrectomy surgery in 1989, the simultaneous performance of these procedures has become increasingly common in treating various retinal and vitreous disorders [1, 2]. This trend is largely due to the accelerated cataract progression following vitrectomy, along with the benefits of improved intraoperative visualization and faster postoperative visual recovery achieved through combined surgery [3, 4]. Moreover, compared to standalone cataract surgery, combined cataract and vitrectomy procedures present additional challenges, including early postoperative intraocular lens (IOL) tilt and decentration, which may result from factors such as vitreous removal and tamponade agents [5–10]. Therefore, achieving proper IOL implantation and ensuring its postoperative stability is crucial in combined cataract and vitrectomy surgeries.

In cataract surgery, factors such as the type of IOL and the success and size of continuous curvilinear capsulorhexis (CCC) play a significant role in the postoperative stability of IOL positioning [11–14]. Specifically, failure to create a CCC with an appropriate size relative to the IOL can result in instability of the lens within the capsular bag [13, 15].

Femtosecond laser‐assisted cataract surgery (FLACS) was introduced in 2010 [16, 17]. This technique enables the creation of a CCC with predetermined size and location, contributing to improved IOL stability [18–20]. However, studies investigating IOL stability in combined FLACS and vitrectomy procedures remain limited [21–23]. Furthermore, none of the studies have specifically investigated the use of 7‐mm IOLs in FLACS combined with vitrectomy surgery. In this study, we evaluated the effects of IOL tilt and decentration following FLACS combined with vitrectomy surgery using 7 mm IOLs.

## 2. Methods

### 2.1. Patients

We reviewed the medical records of patients who underwent combined phacoemulsification and vitreoretinal surgery at the Jikei University Hospital between February 2017 and March 2021. This retrospective study was approved by the Institutional Review Board of the Jikei University School of Medicine and conducted in accordance with the principles of the Declaration of Helsinki (approval number: 32–128 [10204]). Informed consent was obtained from each patient. Data collected included age, sex, decimal best‐corrected visual acuity (BCVA), which was measured using a Landolt C chart and converted to the logarithm of the minimum angle of resolution (logMAR) BCVA, anterior chamber depth (ACD), and IOL tilt and decentration, assessed using swept‐source optical coherence tomography (CASIA2; Tomey Corp., Nagoya, Japan). The inclusion criteria were (1) patients who underwent conventional phacoemulsification surgery combined with vitrectomy or FLACS combined with vitrectomy, (2) in‐the‐bag 7 mm IOL (X70, Santen, Osaka, Japan) implantation, and (3) sufficient quality of preoperative and postoperative data. We excluded patients who experienced severe complications postsurgery and those with toric or multifocal IOL implantation. Patients were divided into four groups depending on the surgical method: conventional phacoemulsification surgery combined with vitrectomy without fluid‐air exchange (F/Ax) (Group A) or with F/Ax (Group B), and FLACS combined with vitrectomy without fluid‐air exchange (F/Ax) (Group C) or with F/Ax (Group D).

### 2.2. Anterior Segment Evaluation

**FIGURE 1:**
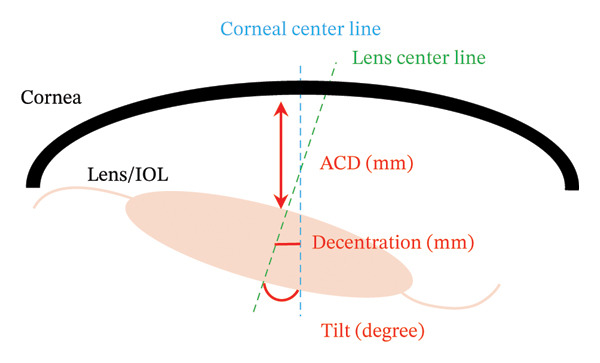
Parameters of swept‐source anterior segment OCT (CASIA2) after cataract surgery and vitrectomy. The IOL tilt is the angle of the lens center line against the corneal center line, and the decentration is the vertical distance between the lens center line and the corneal center line.

The IOL position was assessed at 1 and 7 days and at 1, 3, 6, and 12 months postoperatively, using the CASIA2. Scans were performed on the horizontal axis (0°–180^o^). The scanned images were analyzed using the device’s built‐in software, and ACD and IOL tilt and decentration were obtained. Previous studies have reported methods for measuring the IOL tilt and decentration [24]. Tilt was defined as the angle between the lens axis and the vertex normal, while decentration referred to the vertical distance between the lens center and the vertex normal (Figure 1). The vertex normal was the line connecting the vertex of the corneal topographic map to the fixation point.

### 2.3. Surgical Procedure

In the femtosecond‐laser‐assisted cataract surgery combined with vitrectomy group, a 5.6 mm capsulotomy was created using the CATALYS (Abbott Medical Optics Inc., Santa Ana, CA, USA). And 31 of 54 eyes received lens fragmentation using a quadrant or sextant and softening. In the conventional phacoemulsification surgery combined with vitrectomy group, a CCC measuring approximately 5.0–5.5 mm was manually created using a 26‐gauge bent sharp needle following anterior chamber formation.

In both groups, a 2.4 mm steep corneoscleral incision was made at the 11 o’clock position. Phacoemulsification was performed using the Constellation system (Alcon Laboratories, Inc., Fort Worth, TX, USA), and a 7 mm IOL was implanted in the capsular bag.

**FIGURE 2:**
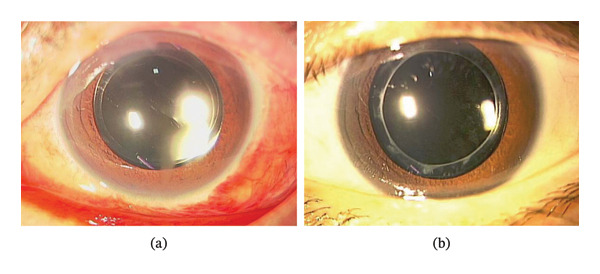
Photographs of the anterior segment. (a) Anterior segment photography after femtosecond laser‐assisted cataract surgery and vitrectomy with gas at postoperative 1 day. The IOL is well centered with a 360° overlapping capsular edge. (b) Anterior segment photography after femtosecond laser‐assisted cataract surgery and vitrectomy with gas at postoperative 12 months. The IOL is well centered with a 360° overlapping capsular edge.

Subsequently, a 25‐gauge or 27‐gauge vitrectomy was performed using the Constellation system, with a wide‐angle viewing system (Resight, Carl Zeiss Meditec AG, Germany). In cases where posterior vitreous detachment had not occurred, it was induced using a vitrectomy cutter. Internal limiting membrane peeling was performed when necessary, and the area was stained with Brilliant Blue G. Depending on the disease, a tamponade with gas (air or 20% SF6) was used, and patients were instructed to maintain a prone position for 1–3 days postoperatively (Figure 2).

### 2.4. Statistical Analyses

Continuous variables are expressed as means ± standard deviations (range). Preoperative and postoperative values (1 day, 1 week, 1 month, 3 months, 6 months, and 12 months) were compared using the Wilcoxon text. The four groups were compared based on the presence or absence of FLACS and gas tamponade using the Kruskal–Wallis test. The significance of the differences among the four groups at each time point was assessed using the Mann–Whitney *U* test. Statistical analyses were performed using Python 3.13.0, scipy.stats module (Python, Wilmington, DE, USA). Results with *p* < 0.01 were considered statistically significant.

## 3. Results

### 3.1. Patients’ Demographics

Patients who underwent combined phacoemulsification using 7 mm IOL (X70) and vitreoretinal surgery at the Jikei University Hospital between February 2017 and March 2021. A total of 153 eyes from 153 patients (47 women and 106 men) were included in this study, with ages 62.3 + 9.5 (range: 37–84) years. The conventional phacoemulsification surgery combined with vitrectomy group consisted of 99 eyes (Groups A and B: 27 and 72 eyes, respectively), while the FLACS combined vitrectomy group consisted of 54 eyes (Groups C and D: 29 and 25 eyes, respectively). Table 1 summarizes clinical diagnoses and demographic characteristics of each group. Complete coverage of the IOL optic by the anterior rhexis margin was achieved in all cases (Table 1).

**TABLE 1:** Patient characteristics.

Group	Overall (A + B)	A	B	Overall (C + D)	C	D
Eye, *n*	99	27	72	54	29	25
Age (years; mean ± SD)	61.0 ± 10.1	69.7 ± 7.6	57.7 ± 8.9	64.7 ± 7.8	65.6 ± 6.9	63.7 ± 8.8
Sex (male/female)	75/24	19/8	56/16	31/23	18/11	13/12
Eye (right/left)	54/45	15/12	39/33	22/32	11/18	11/14
Preoperative						
BCVA (log MAR; mean ± SD)	0.18 ± 0.31	0.31 ± 0.59	0.14 ± 0.27	0.29 ± 0.50	0.28 ± 0.51	0.29 ± 0.48
Postoperative 12 Months						
BCVA (log MAR; mean ± SD)	−0.09 ± 0.54	−0.04 ± 0.36	−0.10 ± 0.57	0.00 ± 0.50	−0.02 ± 0.45	0.05 ± 0.66
AL (mm; mean ± SD)	25.57 ± 1.64	24.50 ± 1.05	25.98 ± 1.64	25.06 ± 1.76	25.18 ± 1.76	24.93 ± 1.80
Preoperative						
ACD (mm; mean ± SD)	2.95 ± 0.33	2.87 ± 0.31	2.98 ± 0.34	2.80 ± 0.38	2.85 ± 0.37	2.74 ± 0.39
Capsulorhexis‐ IOL overlap						
Complete, *n* (%)	100	100	100	100	100	100
Incomplete, *n* (%)	0	0	0	0	0	0
25‐G vitrectomy	98	26	72	53	28	25
27‐G vitrectomy	1	1	0	1	1	0
clinical diagnosis						
Rhegmatogenous retinal detachment	56	0	56	3	0	3
Epiretinal membrane	28	24	4	29	24	5
Macular hole	11	0	11	17	0	17
Proliferative diabetic retinopathy	1	1	0	4	4	0
Vitreous hemorrhage	3	2	1	1	1	0

*Note:* Group A = conventional phacoemulsification surgery combined with vitrectomy without gas, Group B = conventional phacoemulsification surgery combined with vitrectomy with gas, Group C = femtosecond‐laser‐assisted cataract surgery combined with vitrectomy without gas, Group D = femtosecond‐laser‐assisted cataract surgery combined with vitrectomy with gas.

Abbreviations: ACD, anterior chamber depth; AL, axial length; BCVA, best‐corrected visual acuity, IOL, intraocular lens, logMAR = logarithm of the minimum angle of resolution; SD, standard deviation.

### 3.2. ACD Changes

ACD showed significant postoperative changes across all groups compared to the preoperative measurements (*p* < 0.001).

On postoperative Day 1, the ACD in all groups was significantly greater than the preoperative values, indicating an initial deepening of the anterior chamber. In Group A, the mean ACD increased from 2.87 ± 0.31 mm preoperatively to 4.34 ± 0.36 mm on postoperative Day 1. Group C showed a similar trend, with ACD increasing from 2.85 ± 0.37 mm preoperatively to 4.33 ± 0.29 mm. In contrast, Groups B and D exhibited smaller increases on Day 1, with ACD rising from 2.98 ± 0.34 mm to 3.55 ± 0.31 mm in Group B and from 2.74 ± 0.39 mm to 3.34 ± 0.31 mm in Group D. These differences were associated with the presence or absence of gas, while the presence or absence of FLACS did not significantly influence postoperative changes in ACD on postoperative Day 1 (*p* < 0.001).

Postoperatively, distinct trends in ACD were observed among the groups. In Groups A and C, the ACD gradually decreased over time but remained consistently greater than preoperative values, indicating a stable anterior chamber configuration following the initial postoperative deepening. However, the ACD in groups B and D gradually increased over time and stabilized after 1 month postoperatively. At the final follow‐up (12 months postoperatively), the mean ACDs were as follows: 4.25 ± 0.13 mm, 4.23 ± 0.24 mm, 4.25 ± 0.23 mm, and 3.98 ± 0.23 mm in Groups A, B, C, and D, respectively (Table 2).

**TABLE 2:** Anterior chamber depth (mm) outcomes.

Group	Preoperative	1 day	1 week	1 month	3 months	6 months	12 months
A	2.87 ± 0.31	4.34 ± 0.36	4.16 ± 0.28	4.15 ± 0.24	4.17 ± 0.25	4.18 ± 0.16	4.25 ± 0.13
*p* value (pre‐ and postoperative)		< 0.001	< 0.001	< 0.001	< 0.001	< 0.001	< 0.001
B	2.98 ± 0.34	3.55 ± 0.31	4.09 ± 0.30	4.20 ± 0.28	4.21 ± 0.27	4.17 ± 0.24	4.23 ± 0.24
*p* value (pre‐ and postoperative)		< 0.001	< 0.001	< 0.001	< 0.001	< 0.001	< 0.001
C	2.85 ± 0.37	4.33 ± 0.29	4.17 ± 0.23	4.16 ± 0.25	4.18 ± 0.24	4.20 ± 0.25	4.25 ± 0.23
*p* value (pre‐ and postoperative)		< 0.001	< 0.001	< 0.001	< 0.001	< 0.001	< 0.001
D	2.74 ± 0.39	3.34 ± 0.31	3.95 ± 0.33	4.05 ± 0.31	4.07 ± 0.30	4.06 ± 0.30	3.98 ± 0.23
*p* value (pre‐ and postoperative)		< 0.001	< 0.001	< 0.001	< 0.001	< 0.001	< 0.001
*p* value (intergroup)	0.028	< 0.001	0.060	0.298	0.293	0.440	0.063

*Note:* Group A = conventional phacoemulsification surgery combined with vitrectomy without gas, Group B = conventional phacoemulsification surgery combined with vitrectomy with gas, Group C = femtosecond‐laser‐assisted cataract surgery combined with vitrectomy without gas, Group D = femtosecond‐laser‐assisted cataract surgery combined with vitrectomy with gas, pre‐ and postoperative: Wilcoxon test, intergroup: Kruskal–Wallis test.

### 3.3. IOL Tilt Changes

The tilt measurements showed notable differences between the groups both preoperatively and postoperatively. On postoperative Day 1, all groups exhibited an increase in tilt compared to preoperative values. The Kruskal–Wallis test results revealed a significant difference among the groups (*p* < 0.001). In Group D, the mean tilt increased significantly from 4.65° ± 0.95° preoperatively to 7.75° ± 3.30° on postoperative Day 1. Similarly, Group B showed an increase from 4.36° ± 1.83° to 6.66° ± 3.14°. In contrast, the increase was more modest in the groups without gas: Group A increased from 4.41° ± 1.20° to 4.66° ± 1.28°, and Group C showed minimal change, from 3.98° ± 1.60° to 4.00° ± 1.59°.

Postoperative trends varied across the groups. In Group D, the tilt decreased substantially after Day 1 but remained higher than preoperative values, measuring 4.94° ± 1.34° at 1 week and gradually declining to 3.72° ± 1.15° at 12 months. Group B followed a similar pattern, decreasing to 4.90° ± 1.74° at 1 week and stabilizing at 4.18° ± 1.41° at 12 months. Group A demonstrated a gradual decline from 4.66° ± 1.28° on Day 1 to 4.03° ± 1.34° at 12 months. In Group C, tilt values remained relatively stable throughout the postoperative period, with a slight decrease from 4.00° ± 1.59° at Days 1–3.52° ± 1.30° at 12 months (Table 3).

**TABLE 3:** Intraocular lens tilt (°) outcomes.

Group	Preoperative	1 day	1 week	1 month	3 months	6 months	12 months
A	4.41 ± 1.20	4.66 ± 1.28	4.28 ± 1.09	4.45 ± 1.04	4.36 ± 0.98	4.28 ± 1.18	4.03 ± 1.34
*p* value (pre‐ and postoperative)		0.191	0.359	0.860	0.685	0.809	0.138
B	4.36 ± 1.83	6.66 ± 3.14	4.90 ± 1.74	4.25 ± 1.47	4.10 ± 1.48	4.27 ± 1.33	4.18 ± 1.41
*p* value (pre‐ and postoperative)		< 0.001	0.016	0.516	0.233	0.948	0.411
C	3.98 ± 1.60	4.00 ± 1.59	4.04 ± 1.41	4.10 ± 1.70	4.06 ± 1.51	4.07 ± 1.33	3.52 ± 1.30
*p* value (pre‐ and postoperative)		0.711	0.356	0.418	0.611	0.951	0.125
D	4.65 ± 0.95	7.75 ± 3.30	4.94 ± 1.34	4.42 ± 1.18	4.38 ± 1.08	3.89 ± 1.36	3.72 ± 1.15
*p* value (pre‐ and postoperative)		< 0.001	0.218	0.055	0.214	0.090	0.016
*p* value (intergroup)	0.302	< 0.001	0.026	0.715	0.461	0.878	0.436

*Note:* Group A = conventional phacoemulsification surgery combined with vitrectomy without gas, Group B = conventional phacoemulsification surgery combined with vitrectomy with gas, Group C = femtosecond‐laser‐assisted cataract surgery combined with vitrectomy without gas, Group D = femtosecond‐laser‐assisted cataract surgery combined with vitrectomy with gas, pre‐ and postoperative: Wilcoxon test, intergroup: Kruskal–Wallis test.

Using intraoperative gas during pars plana vitrectomy procedures was associated with greater initial postoperative tilt, which subsequently decreased over time. In contrast, cases without gas showed smaller initial changes and more stable tilt measurements during the follow‐up period (Table 3).

### 3.4. IOL Decentration Changes

Decentration measurements varied among the groups both preoperatively and postoperatively.

On postoperative Day 1, all groups exhibited an increase in decentration compared to the preoperative values. The Kruskal–Wallis test results showed a significant difference among the groups on postoperative Day 1 (*p* < 0.001).

In Group B, the mean decentration increased from 0.18 ± 0.10 mm preoperatively to 0.32 ± 0.20 mm on postoperative Day 1. Similarly, Group D showed an increase from 0.15 ± 0.06 mm to 0.30 ± 0.17 mm. In contrast, the groups without gas showed smaller changes: the mean decentration increased from 0.14 ± 0.07 mm to 0.18 ± 0.14 mm in Group A and from 0.17 ± 0.10 mm to 0.19 ± 0.13 mm in Group C (*p* = 0.548, *p* = 0.686).

In Group A, decentration values remained relatively stable postoperatively, measuring 0.18 ± 0.10 mm at 1 week and gradually increasing to 0.25 ± 0.20 mm at 12 months. In Group B, decentration decreased from 0.32 ± 0.20 mm at Day 1 to 0.21 ± 0.14 mm at 1 week and remained consistent around 0.20 ± 0.12 mm at 12 months. In Group C, decentration showed a slight increase to 0.20 ± 0.11 mm at 1 week and stabilized at 0.22 ± 0.13 mm at 12 months. In Group D, decentration decreased from 0.30 ± 0.17 mm at Day 1 to 0.18 ± 0.08 mm at 1 week, followed by a slight increase to 0.24 ± 0.14 mm at 12 months.

The postoperative trends differed among the groups, with the gas groups (Group B and Group D) showing greater initial decentration (*p* < 0.001), which subsequently decreased over time. In contrast, the nongas groups (Groups A and C) exhibited more stable decentration values throughout the follow‐up period (Table 4).

**TABLE 4:** Intraocular lens decentration (mm) outcomes.

Group	Preoperative	1 day	1 week	1 month	3 months	6 months	12 months
A	0.14 ± 0.07	0.18 ± 0.14	0.18 ± 0.10	0.20 ± 0.11	0.19 ± 0.12	0.18 ± 0.13	0.25 ± 0.20
*p* value (pre‐ and postoperative)		0.548	0.452	0.052	0.184	0.600	0.236
B	0.18 ± 0.10	0.32 ± 0.20	0.21 ± 0.14	0.21 ± 0.15	0.22 ± 0.15	0.20 ± 0.12	0.20 ± 0.12
*p* value (pre‐ and postoperative)		< 0.001	0.620	0.615	0.304	0.811	0.633
C	0.17 ± 0.10	0.19 ± 0.13	0.20 ± 0.11	0.21 ± 0.11	0.21 ± 0.12	0.19 ± 0.12	0.22 ± 0.13
*p* value (pre‐ and postoperative)		0.686	0.111	0.030	0.156	0.341	0.210
D	0.15 ± 0.06	0.30 ± 0.17	0.18 ± 0.08	0.20 ± 0.09	0.19 ± 0.11	0.24 ± 0.20	0.24 ± 0.14
*p* value (pre‐ and postoperative)		< 0.001	0.466	0.077	0.643	0.358	0.331
*p* value (intergroup)	0.298	< 0.001	0.797	0.963	0.659	0.915	0.882

*Note:* Group A = conventional phacoemulsification surgery combined with vitrectomy without gas, Group B = conventional phacoemulsification surgery combined with vitrectomy with gas, Group C = femtosecond‐laser‐assisted cataract surgery combined with vitrectomy without gas, Group D = femtosecond‐laser‐assisted cataract surgery combined with vitrectomy with gas pre‐ and postoperative: Wilcoxon test, intergroup: Kruskal–Wallis.

## 4. Discussion

In this study, we compared ACD, IOL tilt, and decentration in the aforementioned four groups based on the type of surgery (FLACS or conventional phacoemulsification) and the use of tamponade agents during vitrectomy. In the groups without tamponade agents, no significant differences were observed in ACD, IOL tilt, or decentration after postoperative Day 1, regardless of the use of a femtosecond laser. Similarly, in the groups with tamponade agents, no significant differences were observed in ACD, IOL tilt, or decentration after postoperative Day 1, irrespective of the use of a femtosecond laser. However, significant differences in ACD and IOL tilt and decentration were observed only on postoperative Day 1 between the gas and nongas groups. This suggests that gas tamponade may transiently affect IOL positioning due to changes in intraocular pressure and posterior pressure. This anterior displacement of the IOL is likely caused by posterior pressure exerted by the tamponade agents. Previous studies have highlighted issues such as IOL tilt and decentration in surgeries involving gas tamponade, which is consistent with our findings of anterior IOL displacement [8, 9].

Postoperative IOL tilt exceeding 7° and decentration > 0.4 mm are critical concerns, as they can compromise IOL performance [25]. In combined cataract and vitrectomy surgeries, it is essential to ensure precise IOL implantation within the capsular bag. Particularly in vitrectomy, vitreous removal and the influence of tamponade agents can destabilize the IOL position. Therefore, creating a CCC that fully covers the intended IOL size is crucial. However, manual CCC creation carries a 0.79% risk of anterior capsule tears, which may compromise IOL stability [26]. Thus, accurate CCC sizing and careful IOL selection are key to the success of combined surgical procedures.

If the capsular bag tears or the CCC extends significantly beyond the IOL edge, the IOL may not be completely covered by the capsular bag, leading to potential instability in the IOL position [11, 13, 15]. In contrast, FLACS allows for the creation of a circular and precisely sized CCC tailored to the intended IOL, offering advantages in ensuring complete coverage [18, 19, 27]. Notably, all cases in our study achieved complete coverage with manually created CCCs by experienced surgeons; however, the possibility of incomplete coverage in a larger sample underscores the superiority of FLACS in ensuring optimal outcomes.

In FLACS combined with gas tamponade, previous reports have demonstrated stable IOL positioning within the capsular bag using CCC sizes of 4.9 mm or 4.8 mm for 6‐mm IOLs on slit‐lamp biomicroscopy [21, 22]. However, these studies did not quantify IOL tilt and decentration. Our study provides valuable insights into postoperative outcomes by quantifying IOL tilt, decentration, and ACD.

Our findings should be interpreted considering the limitations of the study. The sample size was relatively small, and selection bias may be present, as the study was conducted at a single center with patients from a limited geographic region. Another limitation is the lack of functional visual assessments, such as contrast sensitivity testing, which restricted our evaluation to structural outcomes, such as IOL tilt and decentration.

## 5. Conclusions

This study demonstrated that in patients who underwent combined cataract surgery and vitrectomy with planned implantation of a 7 mm IOL, no significant differences were observed in the tilt and decentration of the IOL between 5.6 mm anterior capsulotomy by FLACS and those that received a manual CCC made by an expert surgeon, regardless of gas tamponade. Therefore, in combined phacoemulsification and vitreoretinal surgery with the insertion of a 7 mm IOL, a 5.6 mm capsulotomy created using a femtosecond laser is an equivalent technique to CCC created using conventional cataract surgery because it provides a central circular anterior capsulotomy that completely covers the IOL and stabilizes the position of the IOL in eyes.

## Funding

No funding was received for conducting this study.

## Conflicts of Interest

The authors declare no conflicts of interest.

## Data Availability

The authors have nothing to report.
